# Interstitial atoms enable joint twinning and transformation induced plasticity in strong and ductile high-entropy alloys

**DOI:** 10.1038/srep40704

**Published:** 2017-01-12

**Authors:** Zhiming Li, Cemal Cem Tasan, Hauke Springer, Baptiste Gault, Dierk Raabe

**Affiliations:** 1 Max-Planck-Institut für Eisenforschung, Max-Planck-Straße 1, 40237 Düsseldorf, Germany; 2 Department of Materials Science and Engineering, Massachusetts Institute of Technology, 77 Massachusetts Avenue, Cambridge, MA 02139, USA

## Abstract

High-entropy alloys (HEAs) consisting of multiple principle elements provide an avenue for realizing exceptional mechanical, physical and chemical properties. We report a novel strategy for designing a new class of HEAs incorporating the additional interstitial element carbon. This results in joint activation of twinning- and transformation-induced plasticity (TWIP and TRIP) by tuning the matrix phase’s instability in a metastable TRIP-assisted dual-phase HEA. Besides TWIP and TRIP, such alloys benefit from massive substitutional and interstitial solid solution strengthening as well as from the composite effect associated with its dual-phase structure. Nanosize particle formation and grain size reduction are also utilized. The new interstitial TWIP-TRIP-HEA thus unifies all metallic strengthening mechanisms in one material, leading to twice the tensile strength compared to a single-phase HEA with similar composition, yet, at identical ductility.

Exploring strong and yet ductile materials is paramount for reducing the weight and hence the energy consumption in all fields where mobile structures are used^1^,^2^. However, strength and ductility of current engineering materials are generally conflicting^3^, limiting traditional alloy design strategies. Over the past years, high-entropy alloys (HEAs) have drawn great attention as it opens an entirely new realm of compositional opportunities for designing novel materials with exceptional properties^4^,^5^,^6^,^7^,^8^,^9^,^10^,^11^. HEAs were originally proposed to contain multiple principal elements in near-equimolar ratios to stabilize single-phase solid solutions through maximizing configurational entropy^4^,^5^. Recently, motivated by the fact that maximized configurational entropy is not the sole factor determining phase stability of HEAs^12^,^13^,^14^,^15^,^16^, a novel metastable transformation-induced plasticity dual-phase (TRIP-DP) HEA with exceptional strength and ductility has been developed^6^,^17^. Based on this approach, we propose a new class of HEAs which is interstitially alloyed and unifies all known metallic strengthening mechanisms in one material. We use carbon as interstitial element in line with two main trends which can be deduced from previous studies on advanced steels:

- First, the addition of interstitial carbon into a recently developed TRIP-DP-HEA^6^ leads to an increase in stacking fault energy and hence phase stability^18^. Tuning the stability of the face-centered cubic (f.c.c.) matrix phase in the dual-phase structure to a critical point triggers the twinning-induced plasticity (TWIP) effect while maintaining the TRIP effect, thereby further improving the alloy’s strain-hardening ability^19^,^20^.
- Second, HEAs benefit profoundly from interstitial solid solution strengthening instead of only the established massive solid solution strengthening provided by its multiple principle elements^4^,^5^. This is due to the circumstance that carbon, nitrogen and other interstitials lead to much higher lattice distortions than substitutional elements which strongly affects their interaction with dislocations^21^,^22^.

We produced the interstitial HEA (iHEA) by melting and casting in a vacuum induction furnace using pure metals and carbon with nominal composition Fe_49.5_Mn_30_Co_10_Cr_10_C_0.5_ (at%). The cast alloy was hot-rolled, homogenized and water-quenched. Further grain refinement was achieved by cold-rolling and annealing in Ar atmosphere. The bulk chemical composition of the iHEA measured by wet-chemical analysis is Fe 49.01, Mn 29.87, Co 10.22, Cr 10.34 and C 0.56 (all in at%).

## Results and Discussion

### Coarse-grained iHEA

**Figure 1:**
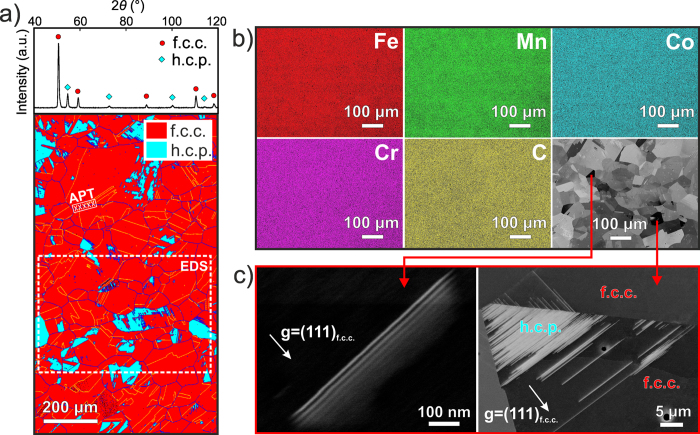
Microstructure and elemental distribution in the as-homogenized coarse-grained iHEA. (**a**) XRD and EBSD patterns reveal that the structure consists of f.c.c. and h.c.p. phases (DP structure). (**b**) EDS maps and BSE images from the region marked in (**a**) show the uniform distributions of all elements at the grain-scale. (**c**) ECCI analysis shows stacking faults and h.c.p. phase within the f.c.c. matrix. Three-dimensional APT tip reconstructions taken from the region marked in (**a**) are given in Supplementary Fig. S1.

Figure 1a shows the X-ray diffraction (XRD) and electron backscatter diffraction (EBSD) patterns of the coarse-grained iHEA after homogenization. The alloy has a dual-phase microstructure consisting of an f.c.c. γ matrix (of ~160 μm grain size) and a laminate hexagonal close-packed (h.c.p.) ε phase (ranging from several nm to 100 μm in thickness). The energy-dispersive X-ray spectroscopy (EDS) maps and the corresponding back-scattered electron (BSE) image in Fig. 1b reveal that all elements (Fe, Mn Co, Cr and C) are uniformly distributed when probed at the grain-scale. The electron channeling contrast imaging (ECCI^23^) analysis in Fig. 1c shows that the stacking faults and h.c.p. phase within the f.c.c. matrix exhibit very similar orientation, which is consistent with previous results, namely, that stacking faults act as nuclei of the h.c.p. phase^6^,^24^. Atom probe tomography (APT) tips were lifted out from a grain boundary region, marked in the EBSD phase map in Fig. 1a using the method outlined in ref. 25. This was to rule out the possibility of nanometer scale elemental partitioning and confirm the distribution of carbon (Supplementary Fig. S1) since the EDS method has a relatively low resolution on light elements. The analysis reveals that the volume probed has an overall composition of Fe_49.63_Mn_27.27_Co_11.65_Cr_10.86_C_0.59_ (at%), showing values consistent with the nominal bulk composition. No apparent elemental segregation was observed (Supplementary Fig. S1), confirming the uniform distribution of all elements also at the nanometer scale. The EDS and APT data also show that both phases benefit from the same level of substitutional and interstitial solid solution content.

### Grain-refined iHEA

**Figure 2:**
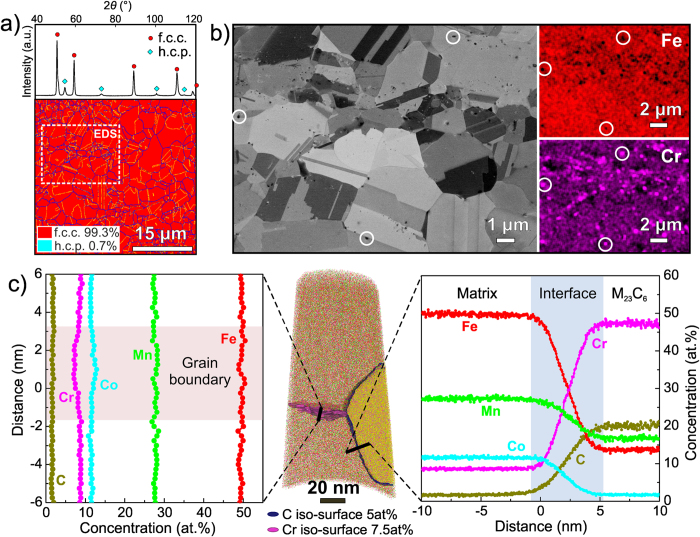
Microstructure and elemental distribution in the grain-refined iHEA. (**a**) XRD and EBSD patterns reveal the f.c.c. matrix and a small fraction of h.c.p. phase prior to deformation. (**b**) ECC image and EDS maps corresponding to the identical region marked in (**a**) show that the nano-sized particles enriched with Cr are randomly distributed in the microstructure. (**c**) APT tip reconstruction revealing elemental distributions across a particle-matrix interface and at an adjacent grain boundary; 5 at% C and 7.5 at% Cr iso-concentration surfaces were used to highlight the nano-carbide and the grain boundary, respectively. The calibration of the APT tip reconstruction based on interplanar spacing is shown in the Supplementary Fig. S2.

After cold-rolling and recrystallization annealing, the average grain size of the iHEA was refined from about 160 μm to approximately 4.0 μm and the h.c.p. phase fraction decreased to 0.7 area % according to EBSD analysis (Fig. 2a). Interestingly, according to the ECC image and EDS maps in Fig. 2b, nano-particles with an average size of 50~100 nm and enriched with Cr but depleted with Fe are found to be randomly distributed in the matrix, rather than clustered at grain boundaries. The chemical compositions of the nano-particles were probed by APT, as shown in Fig. 2c for an interfacial region between a particle and the matrix. The APT data show 47.16 Cr, 17.82 Mn, 13.64 Fe, 1.92 Co and 19.46 C (at%) particle composition, suggesting that they are M_23_C_6_ carbides (M: Cr, Mn, Fe and Co). The f.c.c. structure of these M_23_C_6_ carbides has been confirmed by TEM analysis (see Supplementary Fig. S3). Such nano-sized carbides have the potential to contribute profoundly to the strength of the alloy^26^,^27^. Moreover, the C partition between the matrix and the M_23_C_6_ carbides. The APT data show that ~0.35 at% C is in the matrix, while the carbides have a volume fraction of ~1.5 vol.% as calculated from multiple ECC images. In combination, the C content is consistent with the results of the alloy’s overall C content in the bulk alloy (~0.56 at% obtained by chemical analysis). Additionally, a grain boundary characterized by a slight depletion of Cr was observed adjacent to the nano-carbide as shown in Fig. 2c (left side). This observation indicates that the carbide may preferentially absorb slightly more Cr from the grain boundary than that from the grain interior during nucleation and growth due to faster diffusion along the grain boundary^28^.

### Tensile deformation behavior

**Figure 3:**
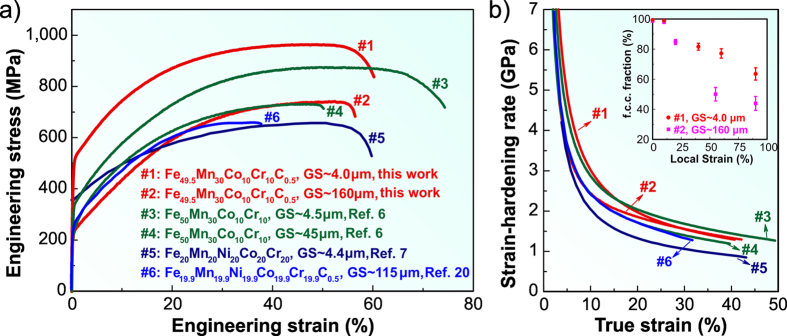
Mechanical behavior of the iHEAs compared to various TRIP-DP-HEAs and single-phase HEAs. GS refers to the grain size. (**a**) Engineering stress-strain curves; data of Fe_50_Mn_30_Co_10_Cr_10_ (at%) TRIP-DP-HEAs (ref. 6), single-phase Fe_20_Mn_20_Ni_20_Co_20_Cr_20_ (at%) and Fe_19.9_Mn_19.9_Ni_19.9_Co_19.9_Cr_19.9_C_0.5_ (at%) HEAs (refs 7 and 20, respectively), are shown for reference. (**b**) Strain-hardening for the same group of alloys. The inset shows the increased stability of the f.c.c. phase upon grain refinement. The data points in the inset are means ± standard deviation for 3 tests.

Figure 3a shows the room-temperature tensile curves of the iHEAs in the coarse-grained (#2, as-homogenized, grain size of ~160 μm) and grain-refined (#1, recrystallized, grain size of ~4.0 μm) states. The curves for the recently developed TRIP-DP-HEAs^6^ in coarse-grained (#4) and grain-refined (#3) states, single-phase equiatomic FeMnCoCrNi HEA^7^ in grain-refined state (#5) and single-phase HEA containing carbon^20^ in coarse-grained state (#6) are presented as reference to underline the improvement of the properties. We observe that even the coarse-grained iHEA (#2, grain size of ~160 μm) shows superior mechanical response, that is, higher elongation and identical ultimate strength compared to the coarse-grained TRIP-DP-HEA (#4, grain size of ~45 μm). Also, it shows significantly higher elongation and ultimate strength compared to the coarse-grained single-phase HEA with the same carbon content (#6, grain size of ~115 μm)^20^. Upon grain refinement, the iHEA (#1) shows substantial improvement in both, yield and ultimate strength, while maintaining the identical elongation compared to its coarse-grained counterpart (#2). Although the grain-refined iHEA (#1) has a slightly lower elongation compared to the grain-refined TRIP-DP-HEA (#3), it shows ~100 MPa (~15%) higher yield and ultimate strength. Also, the tensile strength of the grain-refined iHEA is nearly twice that of the corresponding single-phase equiatomic FeMnCoCrNi alloy, while their elongation values under tensile load are identical.

Figure 3b reveals the corresponding strain-hardening response with respect to the true strain in the various HEAs. All non-equiatomic FeMnCoCr HEAs, both with and without carbon, show higher strain-hardening rate than the corresponding equiatomic FeMnCoCrNi HEA over the entire loading range. During the early stages of deformation, the grain-refined iHEA (#1) exhibits higher strain-hardening than the corresponding grain-refined TRIP-DP-HEA (#3), but it shows slightly lower strain-hardening at later deformation stages. Moreover, the grain-refined iHEA (#1) shows higher strain-hardening than its coarse-grained counterpart (#2), particularly during the early stages of deformation. This is associated with the influence of the grain size on the f.c.c. phase stability (see inset in Fig. 3b) as also observed for a previously developed TRIP-DP-HEA^17^.

### Deformation mechanisms

**Figure 4:**
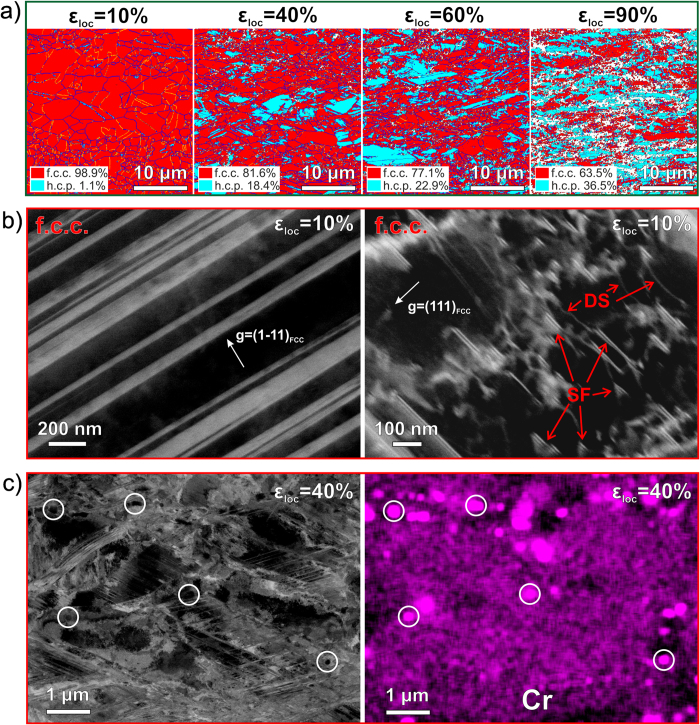
Deformation micro-mechanisms in the grain-refined iHEA with increasing tensile deformation at room temperature. (**a**) EBSD phase maps revealing deformation-induced martensitic transformation as a function of deformation; the local strain (*ε*_loc_) levels of 10%, 40%, 60% and 90% correspond to the early, medium and late uniform deformation and post-necking stages, respectively. (**b**) ECCI analysis revealing deformation induced twins, stacking faults and dislocations in the f.c.c. phase; SF and DS refer to stacking faults and dislocations, respectively. (**c**) ECCI and EDS reveal the presence of carbides enriched with Cr in the microstructure; note that the EDS map (right side) shows a somewhat larger particle size due to image drift during high-magnification long-time EDS mapping; the EDS map of Cr is provided rather than that of C, because EDS has lower resolution on C than on metallic elements.

These improvements in mechanical properties of the iHEA compared to substitutional HEAs are due to the multiple strengthening mechanisms active in the new material. In the following we discuss these mechanisms for the case of the grain-refined iHEA in terms of EBSD (Fig. 4a), ECCI (Fig. 4b) and EDS (Fig. 4c) observations.

The EBSD phase maps (Fig. 4a) show that the metastable f.c.c phase in the iHEA undergoes a strain-induced martensitic transformation from the f.c.c to the h.c.p phase as a primary deformation mechanism affecting the entire bulk matrix. The fraction of transformed h.c.p. phase at the necking stage (*ε*_loc_ = 90%) is 36.5% (in area%), which is much lower than that observed for the substitutional TRIP-DP-HEAs (area% >75%)^6^,^17^. This observation shows that the f.c.c. phase in the iHEA has a higher stability than in the TRIP-DP-HEA^6^. Already during early uniform deformation (*ε*_loc_ = 10%), nano-twinning is observed in the iHEA as an essential additive deformation mechanism (Fig. 4b). In that feature the new material differs from the previously investigated TRIP-DP-HEA, in which nano-twinning occurs only in the h.c.p. phase at later deformation stages^6^. Also, high amounts of stacking faults and dislocations with planar slip behavior are observed even at the early uniform deformation stages (see Fig. 4b). The increased twin and phase boundary density created by these transformation events induces a dynamic microstructure refinement effect providing additional obstacles against dislocation slip, thereby contributing to enhanced strain-hardening^19^. Also, the ECC image and the EDS map reveal nano-carbides in the microstructure (Fig. 4c), suggesting an Orowan strengthening effect due to dislocation bowing around particles^26^,^27^. The deformation micro-mechanisms in the as-homogenized coarse-grained interstitial TWIP-TRIP-HEA are similar as in the grain-refined HEA. The main difference is that the coarse-grained HEA does not show Orowan strengthening (see Fig. 1b and Supplementary Fig. S3).

**Figure 5:**
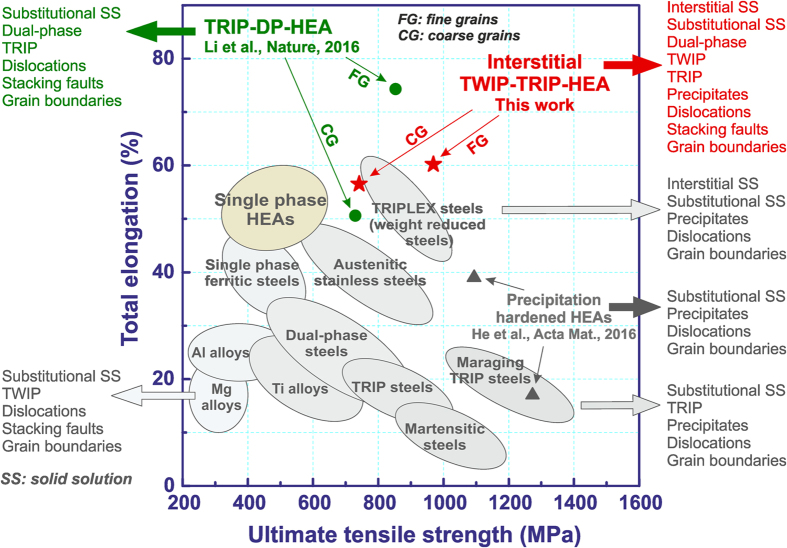
Strength-ductility profiles of various classes of metallic materials including HEAs. All data stem from uniaxial tensile tests conducted on bulk materials at room temperature. While conventional alloys use strengthening mechanisms such as grain boundaries, dual-phase structure, dislocation interactions, precipitates and solid solution (e.g. steels, Ti-alloys, Al- alloys) the new interstitial TWIP-TRIP-HEAs combine all available strengthening effects in one concept, namely, interstitial and substitutional solid solution, TWIP, TRIP, multiple phases, precipitates, dislocations, stacking faults and grain boundaries.

Figure 5 shows the exceptional strength-ductility combination found for interstitial TWIP-TRIP-HEAs. The material exhibits a substantial damage tolerance, characterized here in terms of total elongation multiplied by ultimate tensile strength, exceeding that of most metallic materials. Also, its strength exceeds that of the TRIP-DP-HEAs^6^ and yields significantly higher elongation compared to precipitation hardened HEAs^29^.

The improved mechanical properties (Fig. 3) and the associated multiple deformation mechanisms (Fig. 4) confirm the success of the new design approach, and its potential economic viability since interstitial alloying elements are much cheaper compared to substitutional alloying elements. The new interstitial TWIP-TRIP-HEAs unify all known metallurgical strengthening effects in one single bulk material: i) massive substitutional solid solution strengthening due to multiple principle elements (i.e., Fe, Mn, Co and Cr); ii) interstitial solid solution strengthening by carbon; iii) micro-composite effect from dual-phase structure; iv) nano-particle strengthening from nano-carbides (Figs 2b and 4c); v) nano-twinning induced plasticity effect (TWIP) (Fig. 4b); vi) displacive transformation induced plasticity effect (TRIP) (Fig. 4a); vii) formation of stacking faults (Fig. 4b); viii) grain size reduction (increased grain boundary density); and dislocation hardening (Fig. 4b).

## Conclusions

Our findings demonstrate a new alloy design concept of interstitial TWIP-TRIP-HEAs. The excellent strength-ductility combination of these new materials has very large potential to break the inverse strength-ductility relationship. Also, owing to the unique combination of strengthening mechanisms invoked and the option for interstitial-driven tuning of phase (in)stability further optimization potential is at hand. Indeed, such a broad spectrum of deformation and strengthening mechanisms has never been realized in one single bulk material to date. Also, the interstitial TWIP-TRIP-HEA described here was synthesized by simple addition of interstitial carbon during well-established bulk metallurgical processes available worldwide in metallurgical industries, hence, such alloys can be readily realized in commercial operations.

## Methods

### Materials preparation

The ingots of the interstitial TWIP-TRIP-HEAs with dimensions of 25 × 60 × 65 mm^3^ were first cast in a vacuum induction furnace using pure metals and carbon (>99.8% pure) to predetermined nominal compositions (Fe_49.5_Mn_30_Co_10_Cr_10_C_0.5_, at%). Samples with dimensions of 10 × 25 × 60 mm^3^ machined from the original cast were subsequently hot-rolled at 900 °C to a thickness reduction of 50% (thickness changed from 10 to 5 mm). After hot-rolling, the samples were homogenized at 1200 °C for 2 h in Ar atmosphere followed by water-quenching. To refine the grain size, samples were further cold-rolled to a thickness reduction of 60% and subsequently annealed at the furnace temperature of 900 °C for 3 min in Ar atmosphere followed by water-quenching. Note that the true temperature that the samples actually reached during annealing might be lower than the furnace temperature (900 °C) due to the short annealing time.

### Microstructural and elemental characterization

The microstructures of the alloy in homogenized (coarse-grained) and recrystallized (grain-refined) states were analyzed using various methods. X-ray diffraction (XRD) measurements were performed using an X-Ray equipment ISO-DEBYEFLEX 3003 equipped with Co Kα (λ = 1.788965 Å) radiation operated at 40 kV and 30 mA. Electron backscatter diffraction (EBSD) measurements were carried out by a Zeiss-Crossbeam XB 1540 FIB scanning electron microscope (SEM) with a Hikari camera and the TSL OIM data collection software. Back-scattered electron imaging (BSEI) and electron channeling contrast imaging (ECCI, ref. 23) analyses were performed on a Zeiss-Merlin instrument. The bulk chemical composition of the interstitial TWIP-TRIP-HEA was measured by wet-chemical analysis. The elemental distributions in homogenized and recrystallized alloys were investigated using energy-dispersive X-ray spectroscopy (EDS) and atom probe tomography (APT) (LEAP 3000X HR, Cameca Inc.). Transmission Electron Microscopy (TEM) investigations were conducted on electrochemically prepared samples using a Philips CM20 microscope operated at 200 kV.

### Mechanical characterization

Rectangular dog-bone-shaped specimens for tensile testing, with a thickness of 1 mm, were machined from the alloy sheets in various processing conditions by electrical discharge machining. The gauge length and width of the tensile specimens were 10 and 2.5 mm, respectively. Uniaxial tensile tests were performed using a Kammrath & Weiss tensile stage at an engineering strain rate of 1 × 10^−3^ s^−1^. Groups of three samples for each processing condition were tensile tested at room temperature (293 K). The local strain evolution during tensile test was determined by digital image correlation (DIC) method using Aramis system (GOM GmbH). The deformation microstructures in the fractured tensile samples were investigated by EBSD and ECCI at different regions with different local strain levels.

## Additional Information

**How to cite this article**: Li, Z. *et al*. Interstitial atoms enable joint twinning and transformation induced plasticity in strong and ductile high-entropy alloys. *Sci. Rep.* **7**, 40704; doi: 10.1038/srep40704 (2017).

**Publisher's note:** Springer Nature remains neutral with regard to jurisdictional claims in published maps and institutional affiliations.

## Supplementary Material

Supplementary Information
