# Supplementary material for: Interstitial atoms enable joint twinning and transformation induced plasticity in strong and ductile high-entropy alloys
Source: Sci Rep. 2017 Jan 12;7:40704. doi: 10.1038/srep40704 (PMC5227964; doi:10.1038/srep40704)
Supplement: Supplementary Information [file srep40704-s1.doc]

Supplementary information for

**Interstitial atoms enable joint twinning and transformation induced plasticity in strong and ductile high-entropy alloys**

Zhiming Li^1^, Cemal Cem Tasan^2^, Hauke Springer^1^, Baptiste Gault^1^, Dierk Raabe^1^

^1^Max-Planck-Institut für Eisenforschung, Max-Planck-Straße 1, 40237 Düsseldorf, Germany

^2^Department of Materials Science and Engineering, Massachusetts Institute of Technology, 77 Massachusetts Avenue, Cambridge, MA 02139 USA

Correspondence to: zhiming.li@mpie.de (Z. Li), d.raabe@mpie.de (D. Raabe)

**This part includes:**

Figure S1 to S4


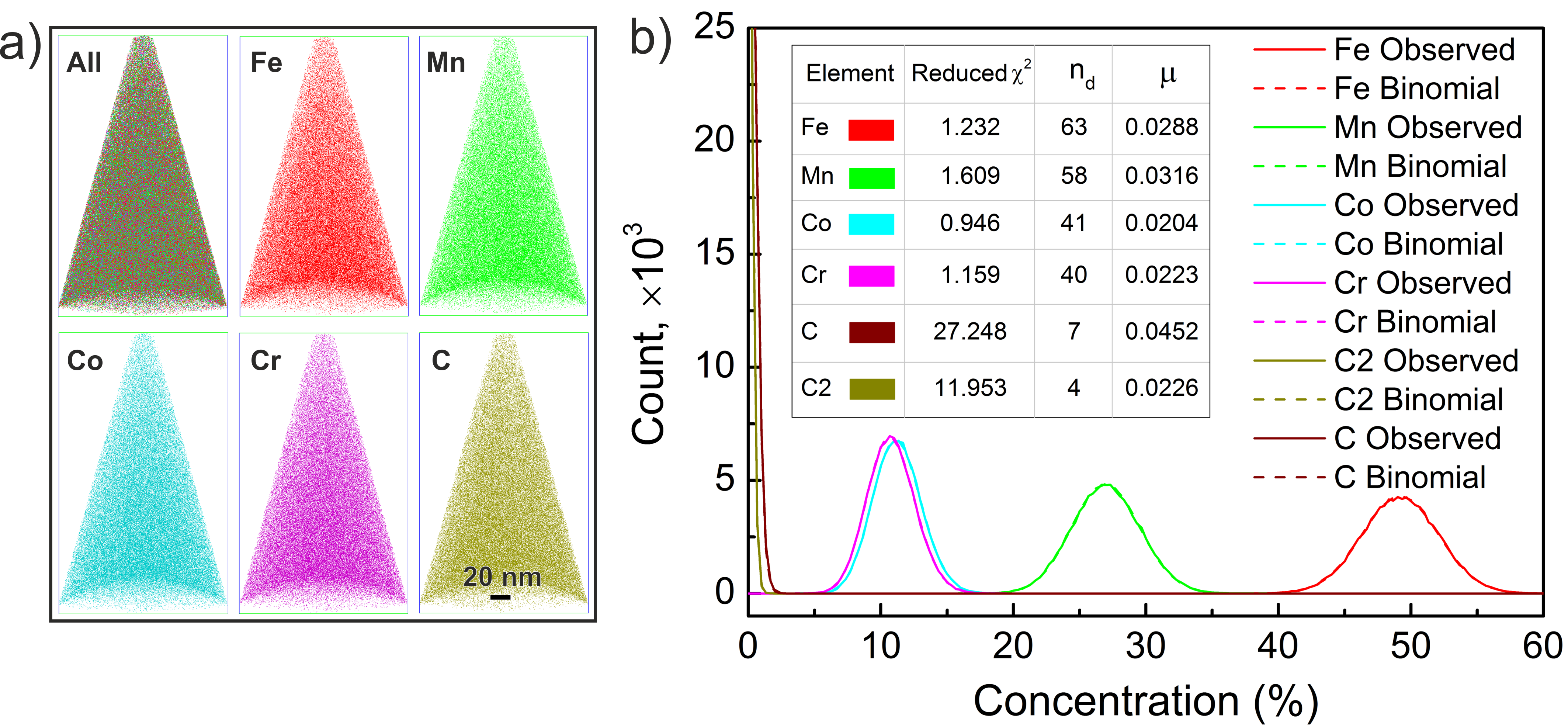


**Figure S1 Typical APT analysis on the coarse-grained interstitial TWIP-TRIP-HEA.** The alloy was homogenized at 1200 ^o^C for 2 h and followed by water-quenching. The region where the APT tips were taken from is marked in the EBSD pattern in Fig. 1a. (**a**) Three-dimensional APT tip reconstructions of atom positions in a typical tip. (**b**) Statistical binomial frequency distribution analysis results showing that the binomial curves obtained from experiments match the curves corresponding to a total random distribution. Several parameters were used to assess the quality of the fit, as listed in the inserted table. n*_d_* and μ are the number of degrees of freedom for a given ion and normalized homogenization parameter, respectively. The values of μ for all elements are close to 0, confirming the random distribution of elements in the coarse-grained interstitial TWIP-TRIP-HEA.


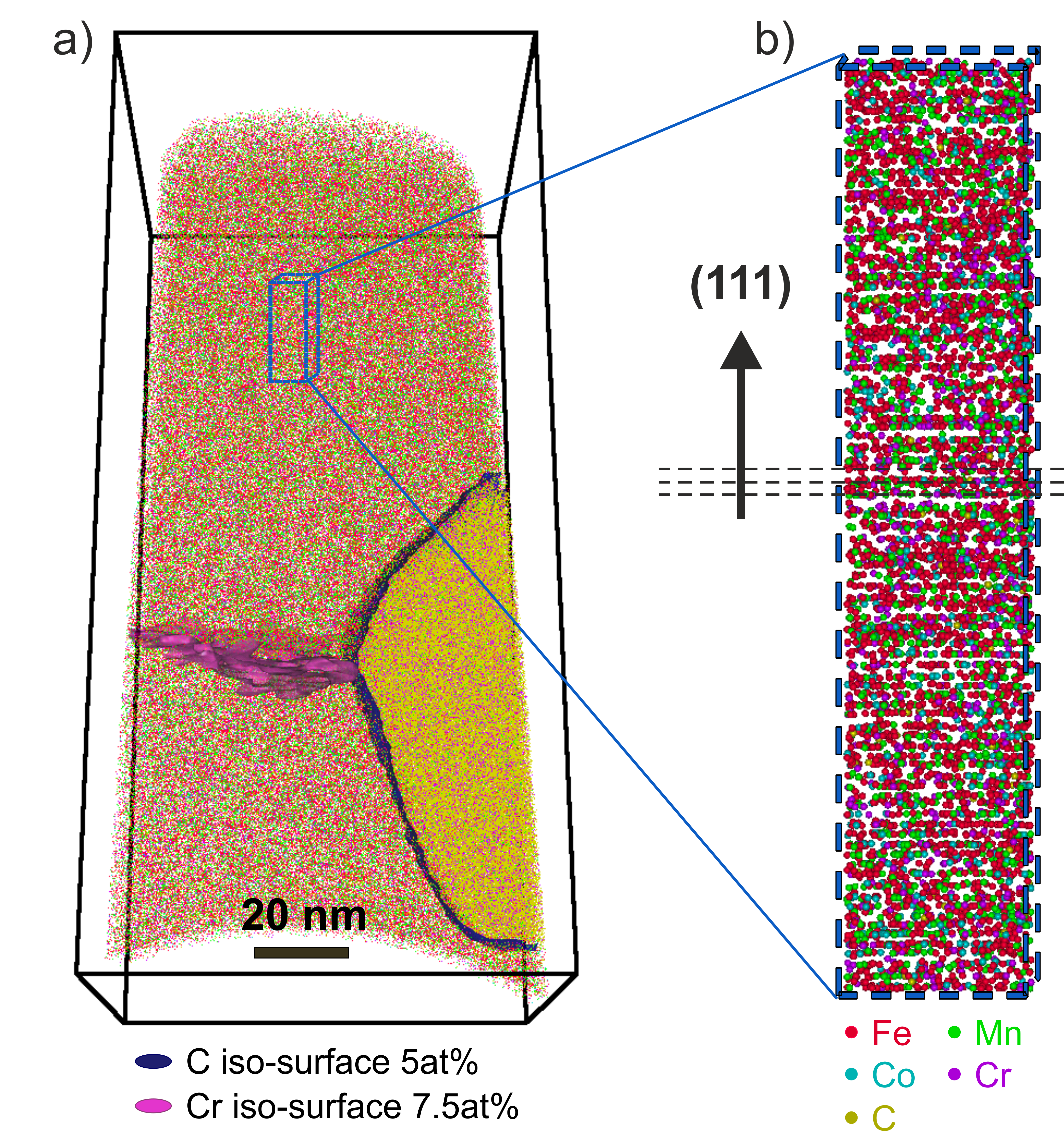


**Figure S2 Three-dimensional APT data set of grain-refined interstitial TWIP-TRIP-HEA.** This data set corresponds to the APT tip shown in Fig. 2c. (**a**) Three-dimensional APT tip reconstruction of all atom positions; 5 at% C and 7.5at% Cr iso-concentration surfaces were used to highlight the nano-carbide and the grain boundary, respectively. (**b**) The atom positions obtained from the magnification of sub-region taken from (**a**) showing that the three-dimensional reconstruction was calibrated based on the (111) interplanar spacing.


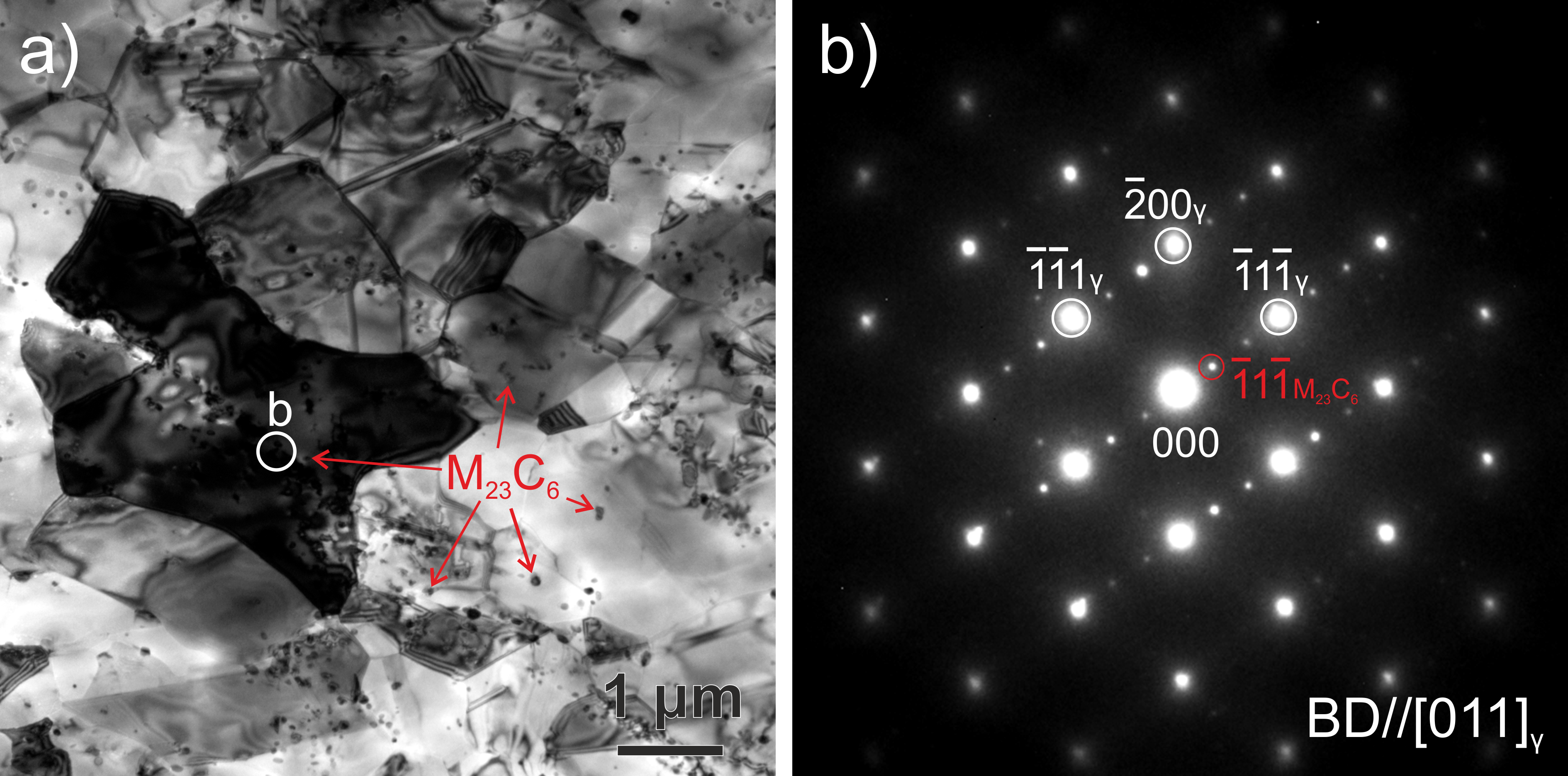


**Figure S3 TEM images of nanosized M_23_C_6_ carbides in the alloy matrix.** (**a**) Bright field image. (**b**) Selected area diffraction pattern taken along the [011] zone axis of the f.c.c. γ matrix confirming the f.c.c. structure of the M_23_C_6_ carbides as well as their cube-on-cube orientation relationship, i.e., [100]γ∥[100]M_23_C_6_ and (100)γ∥(100)M_23_C_6_.


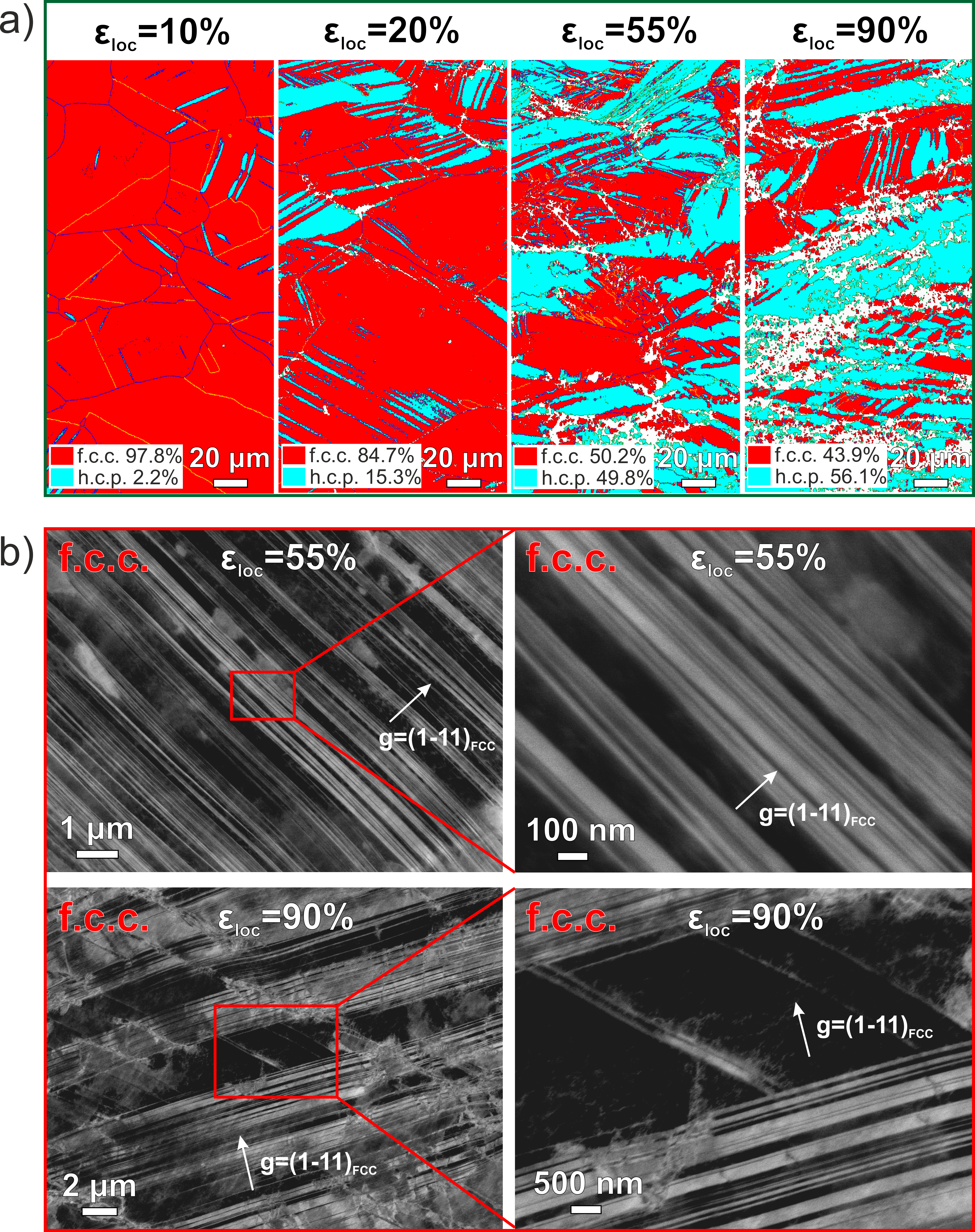


**Figure S4 Deformation micro-mechanisms in the coarse-grained interstitial TWIP-TRIP-HEA with increasing tensile deformation at room temperature.** (**a**) EBSD phase maps showing the deformation-induced martensitic transformation as a function of deformation (TRIP effect). (**b**) ECCI analyses showing the typical microstructure at local strain (*ε*_loc_) levels of 55% and 90%, and revealing the deformation induced twinning in f.c.c. matrix phase (TWIP effect).
